# Microbial Metabolic Genes Crucial for *S. aureus* Biofilms: An Insight From Re-analysis of Publicly Available Microarray Datasets

**DOI:** 10.3389/fmicb.2020.607002

**Published:** 2021-01-28

**Authors:** Rania Nassar, Mahmood Hachim, Mohannad Nassar, Eleftherios G. Kaklamanos, Mohamed Jamal, David Williams, Abiola Senok

**Affiliations:** ^1^College of Medicine, Mohammed Bin Rashid University of Medicine and Health Sciences, Dubai, United Arab Emirates; ^2^School of Dentistry, College of Biomedical and Life Sciences, Cardiff University, Cardiff, United Kingdom; ^3^Department of Preventive and Restorative Dentistry, College of Dental Medicine, University of Sharjah, Sharjah, United Arab Emirates; ^4^Hamdan Bin Mohammed College of Dental Medicine, Mohammed Bin Rashid University of Medicine & Health Sciences, Dubai, United Arab Emirates

**Keywords:** mature biofilm, antibiofilm strategies, biofilm genes, staphylococcal biofilm, *rocD*

## Abstract

Bacterial biofilms are microbial lifestyles found in all environments. Up to 80% of human infections and 60–70% of hospital-acquired infections have a biofilm origin, with *Staphylococcus aureus* one of the leading causes of these infections. Microorganisms in biofilms exhibit significant antimicrobial resistance which poses important treatment challenges, hence the urgent need to identify novel antibiofilm strategies. Microbes form biofilms in response to various factors, and once these 3-dimentional structures form they are highly recalcitrant to removal. The switch from planktonic lifestyle to the biofilm protected mode of growth results in a phenotypic shift in the behavior of the microorganisms in terms of growth rate and gene expression. Given these changes, investigation of microbial gene expression and their modulation at different stages of biofilm maturation is needed to provide vital insight into the behavior of biofilm cells. In this study, we analyzed publicly available transcriptomic dataset of *S. aureus* biofilms at different stages of maturation to identify consistently upregulated genes irrespective of the biofilm maturation stage. Our reanalysis identified a total of 6 differentially expressed genes upregulated in both 48 and 144-h old *S. aureus* biofilms. Functional analysis revealed that these genes encode for proteins which play a role in key microbial metabolic pathways. However, these genes, as yet, are unrelated or fully studied in the context of biofilm. Moreover, the findings of this *in silico* work, suggest that these genes may represent potential novel targets for the development of more effective antibiofilm strategies against *S. aureus* biofilm-associated infections.

## Introduction

Microbial biofilms are ubiquitously found on biotic and abiotic surfaces in environmental, industrial and medical settings (Kostakioti et al., 2013). The cells in these adherent microbial communities are typically embedded in self-produced extracellular polymeric substances (EPS) (Vert et al., 2012). Microbes form biofilms in response to various factors, which can include changes in nutrient availability or exposure of free living (planktonic) cells to sub-inhibitory concentrations of antibiotics (De la Fuente-Núñez et al., 2013). Biofilm development on biotic surfaces follows a universal ordered sequence of events, initiated by adherence of planktonic microorganisms to the surfaces via surface charges and specific adhesin-receptor interactions (Hinsa et al., 2003). Sustained adherence triggers a switch to the biofilm protected mode of growth, which results in a phenotypic shift in the behavior of the microorganisms in terms of growth rate and gene expression (Armbruster and Parsek, 2018). Following irreversible attachment, cells multiply and start to produce biofilm matrix components, leading to small aggregates of microorganisms called microcolonies which eventually develop into large cellular aggregates encased in the self-produced EPS. Once these 3-dimentional structures form they are highly recalcitrant to removal.

It is estimated that up to 80% of human infections (Römling and Balsalobre, 2012) and 60–70% of hospital-acquired infections have a biofilm origin, with *Staphylococcus aureus* one of the leading causes of such infections (Haney et al., 2018). It has been shown that microorganisms within biofilms are protected from both host defenses and administered antimicrobials, with up to 1,000-fold greater tolerance to certain antimicrobials reported (Davies, 2003), which accounts for frequent treatment failures associated with biofilm infections. Hence, there is an urgent need for the development of novel antibiofilm agents and strategies.

Given the changes in microbial gene expression that occur during the biofilm lifestyle, investigation of microbial gene expression and their modulation at different stages of biofilm maturation is needed to provide vital insight into the behavior of biofilm cells. This will indeed help in the identification of genes necessary for biofilm survival, and thus identifying attractive targets for antibiofilm strategies. Using publicly available transcriptomic datasets, this study was undertaken to identify consistent differentially expressed genes (DEGs) in *S. aureus* biofilms at different stages of maturation. To the best of our knowledge, this is the first reported re-analysis of publicly available *S. aureus* transcriptomic datasets for biofilm related gene expression and the findings demonstrate prospective genes and pathway associations that may be targeted for antibiofilm therapies.

## Methods

### Bacterial Transcriptomic Profiling Dataset

**Figure 1:**
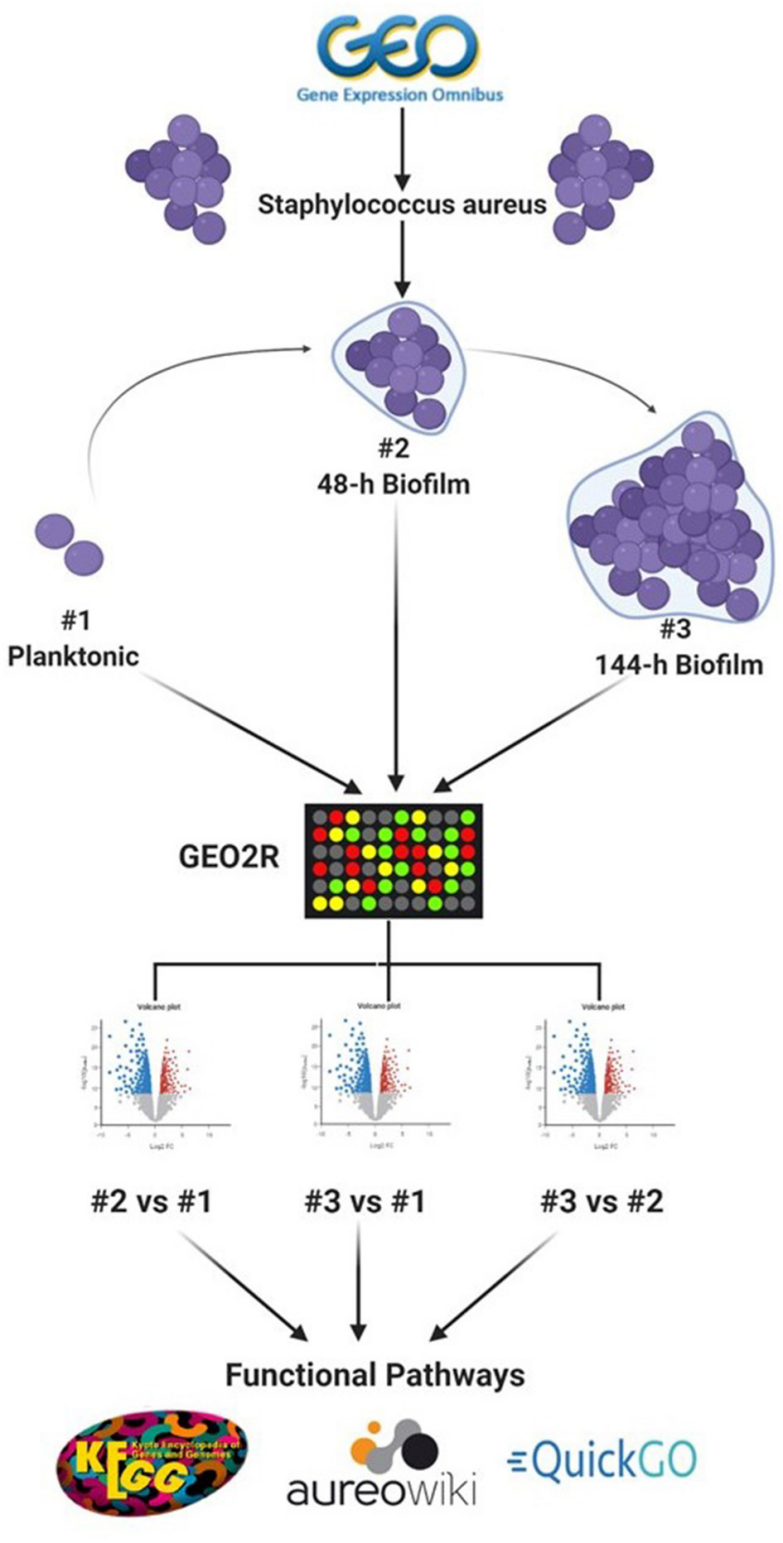
Schematic representation of the reanalysis process of publicly available dataset (GSE35837) retrieved from GEO Omnibus database (https://www.ncbi.nlm.nih.gov/geo/). Created with Biorender.com.

To identify datasets for assessment of DEGs in *S. aureus* biofilms, the data repository of the National Center for Biotechnology Information (NCBI), Gene Expression Omnibus (GEO) (https://www.ncbi.nlm.nih.gov/geo/), was searched for datasets that include *in vitro* planktonic cultures and biofilm cultures at different maturation stages. The single dataset (Accession number: GSE35837) which fulfilled this inclusion criteria was derived from *S. aureus* at three growth stages (planktonic, 48-h and 144-h biofilm), with three replicates for each growth stage (Ryder et al., 2012). Figure 1 shows simplified flowchart of the reanalysis process.

### Identification of DEGs

**Figure 2:**
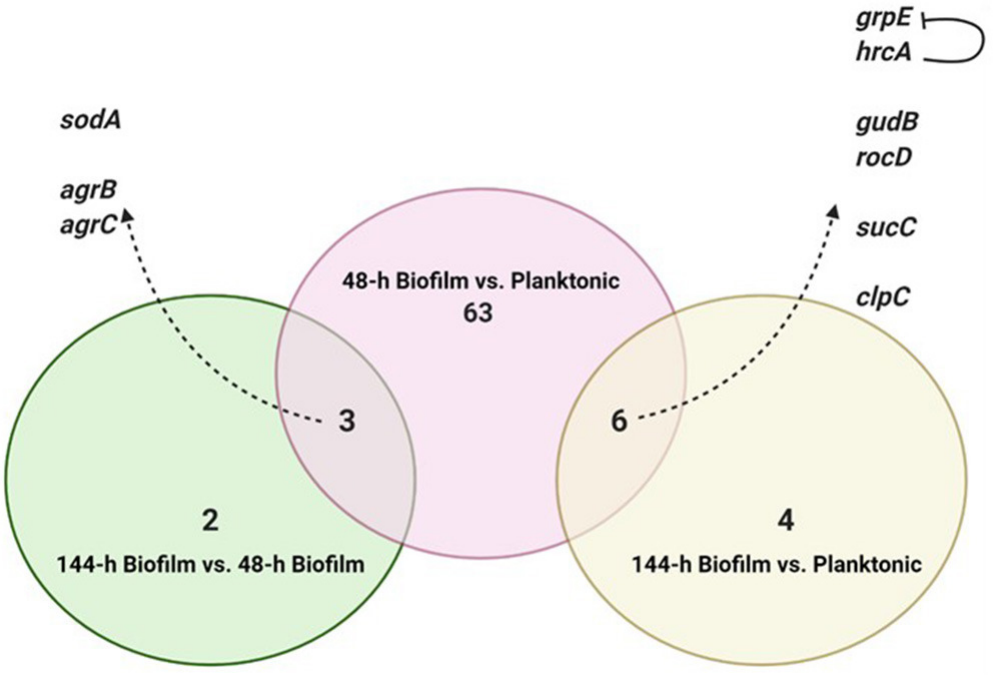
Intersection of differentially expressed genes from the three comparisons. Created with Biorender.com.

To identify DEGs between the three growth stages, GEOquery and limma R packages were used through the GEO2R tool for each comparison, as previously described (Barrett et al., 2013; Hachim et al., 2020). Briefly, the top 5,000 probes with an adjusted *p* < 0.1 were selected as the differentially expressed probes in each of three comparisons (48-h old biofilm vs. planktonic, 144-h old biofilm vs. planktonic and 144-h old biofilm vs. 48-h old biofilm). To identify consistent DEGs in biofilm irrespective of the stage of biofilm maturation, the annotated genes of each comparison were intersected as shown in Figure 2.

### Gene Ontology and Pathway Analysis

Publicly available domains: quickGO, aureuwiki and kyoto encyclopedia of genes and genomes (KEGG) were searched to identify the biological role and functional pathways associated with the consistently DEGs.

## Results

Our re-analysis selection criteria for data curation resulted in one dataset: GSE35837. Comparative analysis of *S. aureus* 48-h old biofilm growth state vs. planktonic cells in the dataset yielded 72 DEGs. Comparison between *S. aureus* 144-h old biofilm growth state vs. planktonic cells yielded 10 DEGs. While from the comparison of 144-h old biofilm and 48-h old biofilm, 5 DEGs were identified. The full list of all DEGs identified is shown in the Supplementary Tables 1–3.

To identify consistent DEGs in the two biofilm maturation stages, the annotated genes of each comparison were intersected (Figure 2). Nine genes were found to be deferentially expressed in both 48-h and 144-h old biofilms. Of these 9 DEGs, 6 were upregulated in both biofilm growth stages (Figure 2). Functional pathway analysis showed that these 6 DEGs include genes involved in cellular amino acid metabolic process (*rocD* and *gudB*), energy metabolism/tricarboxylic acid (TCA) cycle (*sucC*), and stress responses (*grpE, hrcA*, and *clpC*) (Table 1). Three genes were found to be up-regulated in the 48-h biofilm, but down-regulated in 144-h biofilm. These included two accessory regulatory genes (*agrB, agrC*) and *sodA* (Table 1).

**Table 1:** Differentially expressed genes present in both biofilm growth stages (48 and 144-h), the biological processes they are involved in and their associated functional pathways.

**Gene ID**	**Gene name**	**Expression in 44**	**Expression in 144**	**GO Biological processes**	**Pathway**
SAOUHSC_00895	*gudB*	Upregulated	Upregulated	**GO:0006538** glutamate catabolic process**GO:0055114** oxidation-reduction process**GO:0006520** cellular amino acid metabolic process**GO:0006561** proline biosynthetic process	**sao00220** Arginine biosynthesis (KEGG) **sao00430** Taurine and hypotaurine metabolism (KEGG) **sao00910** Nitrogen metabolism (KEGG) **sao01100** Metabolic pathways (KEGG) **sao01120** Microbial metabolism in diverse environments (KEGG)
SAOUHSC_00894	*rocD*	Upregulated	Upregulated	**GO:0006561** proline biosynthetic process**GO:0055129** L-proline biosynthetic process**GO:0008652** cellular amino acid biosynthetic process	**sao00330** Arginine and proline metabolism (KEGG) **sao01100** Metabolic pathways (KEGG) **sao01110** Biosynthesis of secondary metabolites (KEGG)
SAOUHSC_01684	*grpE*	Upregulated	Upregulated	**GO:0006457** protein folding**GO:0050790** regulation of catalytic activity	Stress response/heat shock (aureuwiki)
SAOUHSC_01685	*hrcA*	Upregulated	Upregulated	**GO:0045892** negative regulation of transcription, DNA-templated**GO:0006355** regulation of transcription, DNA-templated**GO:0045892** negative regulation of transcription, DNA-templated	Stress response/heat shock (aureuwiki)
SAOUHSC_01216	*sucC*	Upregulated	Upregulated	**GO:0006099** tricarboxylic acid cycle**GO:0006104** succinyl-CoA metabolic process	**sao00020** Citrate cycle (TCA cycle) (KEGG) **sao00640** Propanoate metabolism (KEGG) **sao00660** C5-Branched dibasic acid metabolism (KEGG) **sao01100** Metabolic pathways (KEGG) **sao01110** Biosynthesis of secondary metabolites (KEGG) **sao01120** Microbial metabolism in diverse environments (KEGG) **sao01200** Carbon metabolism (KEGG)
SAOUHSC_00505	*clpC*	Upregulated	Upregulated	**GO:1990170** stress response to cadmium ion**GO:1990169** stress response to copper ion**GO:0009405** pathogenesis	Protein degradation Proteolysis in bacteria, ATP-dependent (aureuwiki)
SAOUHSC_01653	*sodA*	Upregulated	Downregulated	**GO:0006801** superoxide metabolic process**GO:0055114** oxidation-reduction process**GO:0019430** removal of superoxide radicals	Stress response/Oxidative stress (aureuwiki)
SAOUHSC_02261	*agrB*	Upregulated	Downregulated	**GO:0009405** pathogenesis**GO:0009372** quorum sensing**GO:0006508** proteolysis	**sao02020** Two-component system (KEGG) **sao02024** Quorum sensing (KEGG)
SAOUHSC_02264	*agrC*	Upregulated	Downregulated	**GO:0000160** phosphorelay signal transduction system**GO:0023014** signal transduction by protein phosphorylation	**sao02020** Two-component system (KEGG) **sao02024** Quorum sensing (KEGG)

## Discussion

**Figure 3:**
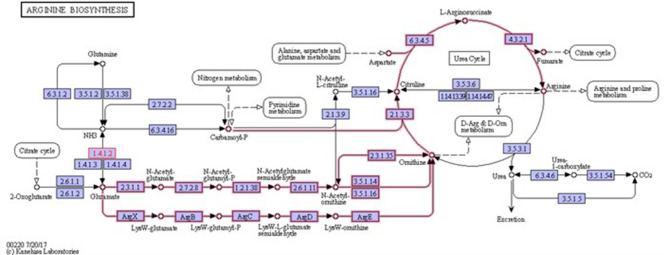
Arginine biosynthesis. Highlighted pathways show the involvement of glutamate in ornithine and arginine biosynthesis. It also shows the involvement of ornithine in arginine biosynthesis. 1.4.1.2 is glutamate dehydrogenase enzyme that is involved in glutamate metabolism (source: KEGG).

**Figure 4:**
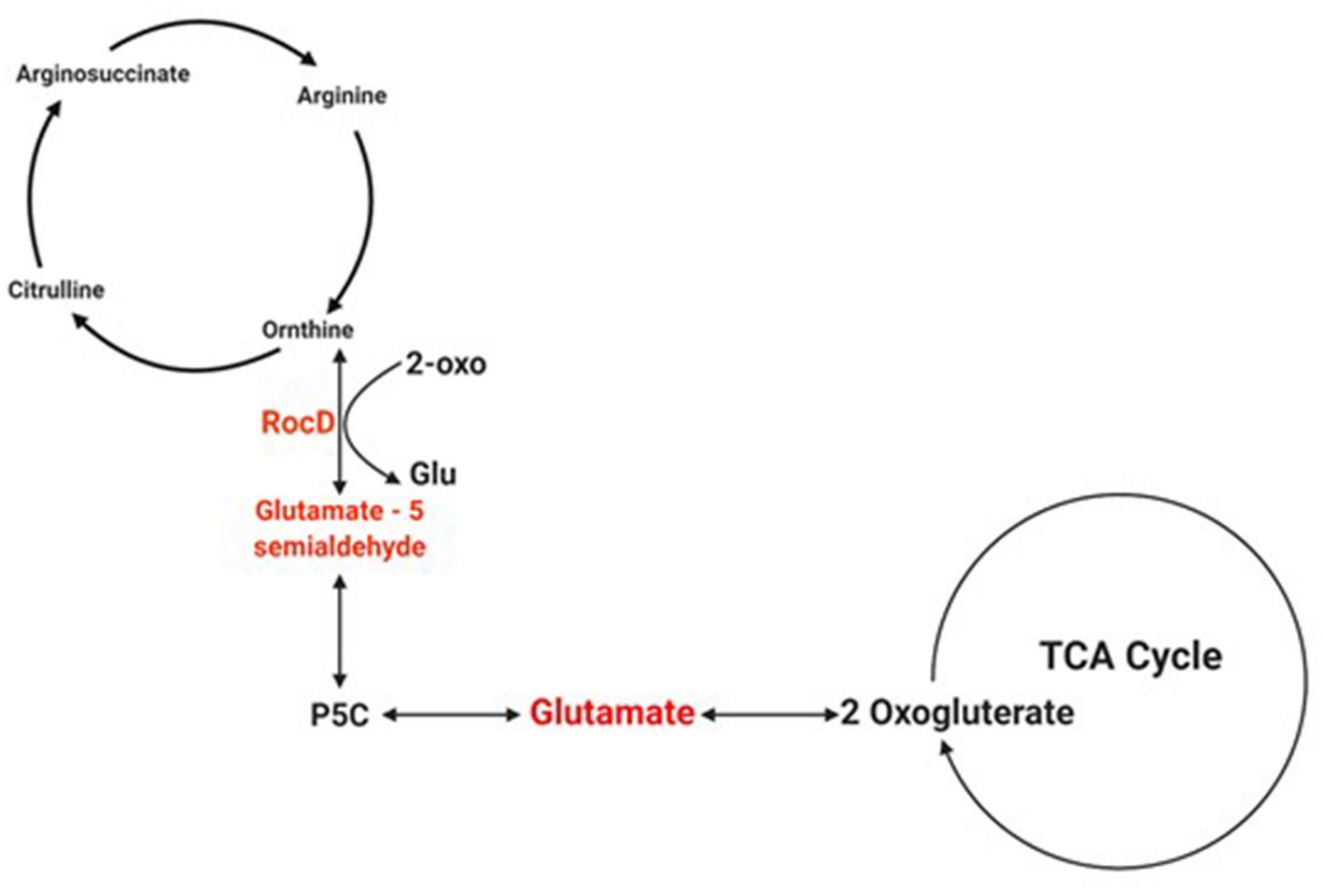
Schematic of the pathway of glutamate involvement in the TCA cycle. The schematic shows how the product of *rocD* converts ornithine to glutamate 5-semialdehyde, and how the latter is involved in glutamate synthesis. Created with Biorender.com.

Biofilms contribute to a large proportion of hospital-acquired infections with *S. aureus* a key aetiological agent (Lister and Horswill, 2014). The stages of biofilm formation from attachment, growth, maturation, and the maintenance of the mature biofilm involve multiple complex pathways (O'Toole et al., 2000; Hinsa et al., 2003). Hence, the bacterial cells within a biofilm undergo gene expression and transcriptional changes in response to stressors, and metabolic and quorum sensing requirements for the different stages of biofilm growth (Whiteley et al., 2001; Schembri et al., 2003; Beloin et al., 2004; Vuong et al., 2004a). The understanding of these genetic modulations within the microbial biofilm and the key genes that play a role in both early and mature biofilms have yet to be fully elucidated. Due to the poor efficacy of currently used antimicrobial agents, the identification of novel antibiofilm targets to combat these infections is crucial. Therefore, an understanding of these genetic modulations might provide insight into novel antibiofilm targets. To address this gap in the literature, a re-analysis of publicly available transcriptomic data for *S. aureus*, and compared the changes in gene expression between *S. aureus* planktonic cells and their biofilm counterparts at different maturation stages was undertaken. Six genes namely *rocD, gudB, sucC, grpE, hrcA*, and *clpC* were upregulated at both biofilm growth stages. This suggested that these six genes played a crucial role in different biofilm maturation stages. Two of these genes, *rocD* and *gudB*, are involved in amino acid metabolism, specifically arginine metabolism and biosynthesis. *rocD* and *gudB* encode for ornithine-oxo-acid transaminase and glutamate dehydrogenase, respectively. The glutamate dehydrogenase enzyme is involved in glutamate, glutamine and ammonia metabolism (Figure 3). It has been shown that glutamine and glutamate are essential for the development and survival of *Bacillus subtilis, Enterococcus faecalis*, and *Pseudomonas aeruginosa* biofilms, and interference with their metabolism has detrimental effects on the biofilm. However, their specific contribution to biofilm development is yet to be determined (Hassanov et al., 2018). In a study using an *in vivo* hamster model, a mutant *Clostridium difficile* that was defective for the gene that encodes glutamate dehydrogenase, was unable to successfully colonize (Girinathan et al., 2016). To the best of our knowledge, no studies have been done to investigate the role of *gudB* product in the development and fitness of *S. aureus* biofilm. Of note, glutamate is also involved in ornithine and arginine biosynthesis (Figure 3) and the TCA cycle (Figure 4). *rocD* encodes for ornithine-oxo-acid transaminase enzyme which catalyses interconversion of ornithine to glutamate 5-semialdehyde (Figure 4). Both ornithine and glutamate 5-semialdehyde are involved in glutamate and arginine metabolism (Figures 3, 4). The role of arginine in biofilm development and pH homeostasis has previously been highlighted (Crow and Thomas, 1982; Liu et al., 1995; Zhu et al., 2007; Lindgren et al., 2014). Its role as an energy source in the absence of glucose was also investigated and it was shown that arginine, in addition to other amino acids, acts as a fuel for glutamate synthesis, in which the latter will serve as a source of carbon for the TCA cycle (Halsey et al., 2017). Therefore, given that *rocD* and *gudB* are involved in pathways of metabolites that are shown to have cardinal roles in biofilm development, we hypothesize that interference in their synthesis and use could potentially have deleterious effects on biofilm, thus making them potential targets for antibiofilm strategies. Experimental work using inhibitors of glutamine and glutamate synthesis can be used to further explore this premise (Arkin and Grossowicz, 1970; Hassanov et al., 2018; Nadar et al., 2019). To the best of our knowledge, no investigations have been done on the specific contribution of ornithine-oxo-acid transaminase enzyme on biofilm development and its metabolic contribution to biofilm development remains to be determined.

*sucC* was also upregulated in both 48-h and 144-h biofilms. The product of *sucC* is involved in energy metabolism via the TCA cycle. The upregulation of the TCA cycle under conditions of oxygen depletion enables better use of traces of oxygen, metabolism of excreted fermentation products like acetic or lactic acid, and catabolism of amino acids to meet the microbial need and enhance virulence (Zhu et al., 2009; Gaupp et al., 2010). Bacterial biofilms are formed in response to adverse environmental conditions including oxygen depletion and nutrient deprivation and the upregulation of this gene will be crucial to the maintenance of the biofilm lifestyle. Indeed, in a previous work, a significantly higher amount of dead cells was found in the biofilms formed by *sucC* mutants which is indicative of a role for *sucC* in biofilm maintenance (De Backer et al., 2018). Furthermore, the universal heat-shock response is also a protective mechanism that is vital for bacterial survival and adaptation in these adverse conditions. The *grpE* and its negative regulator *hrcA*, which are part of heat-shock genes (Roncarati and Scarlato, 2017), are also found to be up-regulated in both biofilm growth stages. The product of *grpE* plays an important role in bacterial response during environment stress by preventing the aggregation of stress-denatured proteins (Roncarati and Scarlato, 2017). Following bacterial adaptation to the stressors, the quantity of heat-shock proteins typically decreases to the steady-state needed for continued support of bacterial growth in the stressed environment (Schumann, 2016). This process is in keeping with our finding of the concomitant up-regulation of *hrcA*, which is a negative regulator for *grpE*. Both the TCA cycle and heat-shock response are crucial for overall bacterial survival and fitness which further makes these genes attractive potential antibiofilm targets as it could be postulated that the bacteria are unlikely to make genetic changes to them. As the specific role of these three genes in biofilm development, survival or fitness has hitherto not been reported, further experimental work is warranted.

Previous reports have shown that expression of *clpC* is upregulated in *S. aureus* biofilms compared to planktonic controls (Becker et al., 2001; Resch et al., 2006). This gene encodes the ClpC ATPase that has multiple functions including participation in the degradation of misfolded proteins. Frees et al. (2004) also showed that ClpC was required for *S. aureus* growth under thermal stress and was also required for biofilm formation (Frees et al., 2004). It also acts as ATPase partner of ClpP protease, the product of *clpP* that was shown to be essential for biofilm formation in *Pseudomonas fluorescens clpC*. Our findings further indicate the role of this *clpC* in different stages of biofilm formation, making it a potential antibiofilm target.

Superoxide dismutase converts deleterious superoxide to oxygen and hydrogen peroxide, and the latter is metabolized by catalase. *sodA* which encodes superoxide dismutase was upregulated in 48-h and down regulated in 144-h old biofilm. Similar findings were also demonstrated for the genes of the Agr quorum-sensing regulator system (*agrB* and *agrC*). Although the Agr system plays a role in the development of the biofilm architecture and structure, it has been shown that its sustained expression results in up-regulation of proteases which inhibit biofilm formation and contributes to biofilm dispersion (Boles and Horswill, 2008; Lauderdale et al., 2009; Otto, 2013). Thus, down-regulation of Agr leads to extensive biofilm maturation and enhanced bacterial colonization (Vuong et al., 2004b). Indeed, targeting these genes via the use of Agr-inhibitors on biofilm-associated staphylococcal infections has shown to have counterproductive effects (Vuong et al., 2004b). Thus, given the discrepant gene expression between biofilm growth stages these three genes (*sodA, agrB*, and *agrC*) might appear less attractive as antibiofilm targets when compared to the other genes we identified. However, it is still important to investigate them further in validation experiments.

In conclusion, we have identified six core genes crucial for mature *S. aureus* biofilms. As these genes encode for proteins which play a role in key microbial metabolic pathways, they represent potential novel targets for antibiofilm strategies. Experimental work to validate these findings is strongly recommended as this will be crucial for exploring any translational potential. In addition, it will be of interest to explore the contribution of host factors to biofilm formation and gene expression.

## Data Availability Statement

Publicly available datasets were analyzed in this study. This data can be found here: (https://www.ncbi.nlm.nih.gov/geo/ (Accession number: GSE35837).

## Author Contributions

All authors listed have made a substantial, direct and intellectual contribution to the work, and approved it for publication.

## Conflict of Interest

The authors declare that the research was conducted in the absence of any commercial or financial relationships that could be construed as a potential conflict of interest.
