# Supplementary material for: Microbial Metabolic Genes Crucial for S. aureus Biofilms: An Insight From Re-analysis of Publicly Available Microarray Datasets
Source: Front Microbiol. 2021 Jan 28;11:607002. doi: 10.3389/fmicb.2020.607002 (PMC7876462; doi:10.3389/fmicb.2020.607002)
Supplement: Supplementary file 1 [file Data_Sheet_1.docx]

| **Table S1: DEGs between 48 hr biofilm growth vs. planktonic** | | | |
| --- | --- | --- | --- |
| GENE_INFO | logFC | P.Value | adj.P.Val |
| 1. CDS\|GI:88194651;GeneID:3921740\|GenBank\|0904\|NC_007795\|857718-858908\|ornithine--oxo-acid transaminase\|ornithine aminotransferase, putative\|Staphylococcus aureus subsp. aureus NCTC 8325, complete genome.\|SAOUHSC_00894 | 1.383387 | 1.32E-05 | 0.011388 |
| 1. CDS\|GI:88194652;GeneID:3921741\|GenBank\|0904\|NC_007795\|859017-860261\|glutamate dehydrogenase, NAD-specific, putative\|\|Staphylococcus aureus subsp. aureus NCTC 8325, complete genome.\|SAOUHSC_00895 | 1.236634 | 1.38E-05 | 0.011753 |
| 1. CDS\|GI:88195361;GeneID:3920105\|GenBank\|0904\|NC_007795\|1567139-1567738\|superoxide dismutase, Mn, putative\|\|Staphylococcus aureus subsp. aureus NCTC 8325, complete genome.\|SAOUHSC_01653 | 1.467456 | 2.63E-05 | 0.012117 |
| 1. CDS\|GI:88195932;GeneID:3919137\|GenBank\|0904\|NC_007795\|2093874-2094443\|accessory gene regulator protein B\|\|Staphylococcus aureus subsp. aureus NCTC 8325, complete genome.\|SAOUHSC_02261 | 1.463279 | 2.93E-05 | 0.012168 |
| 1. CDS\|GI:88194954;GeneID:3919483\|GenBank\|0904\|NC_007795\|1168074-1168982\|succinyl-CoA synthetase subunit alpha\|Catalyzes the only substrate-level phosphorylation in the TCA cycle\|Staphylococcus aureus subsp. aureus NCTC 8325, complete genome.\|SAOUHSC_01218 | 0.829728 | 7.19E-05 | 0.015753 |
| 1. CDS\|GI:88194792;GeneID:3919888\|GenBank\|0904\|NC_007795\|1008407-1009519\|pyruvate dehydrogenase complex, E1 component, alpha subunit, putative\|\|Staphylococcus aureus subsp. aureus NCTC 8325, complete genome.\|SAOUHSC_01040 | 0.896555 | 7.89E-05 | 0.016268 |
| 1. CDS\|GI:88194793;GeneID:3919889\|GenBank\|0904\|NC_007795\|1009523-1010500\|pyruvate dehydrogenase complex, E1 component, pyruvate dehydrogenase beta subunit, putative\|\|Staphylococcus aureus subsp. aureus NCTC 8325, complete genome.\|SAOUHSC_01041 | 0.978416 | 0.000105 | 0.017468 |
| 1. CDS\|GI:88195934;GeneID:3919139\|GenBank\|0904\|NC_007795\|2094653-2095897\|accessory gene regulator protein C\|\|Staphylococcus aureus subsp. aureus NCTC 8325, complete genome.\|SAOUHSC_02264 | 1.387413 | 0.000128 | 0.019362 |
| 1. CDS\|GI:88194953;GeneID:3919482\|GenBank\|0904\|NC_007795\|1166886-1168052\|succinyl-CoA synthetase subunit beta\|catalyzes the interconversion of succinyl-CoA and succinate\|Staphylococcus aureus subsp. aureus NCTC 8325, complete genome.\|SAOUHSC_01216 | 1.285021 | 0.000135 | 0.02134 |
| 1. CDS\|GI:88194849;GeneID:3920745\|GenBank\|0904\|NC_007795\|1064670-1065284\|succinate dehydrogenase cytochrome b-558 subunit, putative\|\|Staphylococcus aureus subsp. aureus NCTC 8325, complete genome.\|SAOUHSC_01103 | 1.112999 | 0.000207 | 0.02455 |
| 1. CDS\|GI:88193827;GeneID:3919177\|GenBank\|0904\|NC_007795\|3912-5024\|recombination protein F\|Required for DNA replication; binds preferentially to single-stranded, linear DNA\|Staphylococcus aureus subsp. aureus NCTC 8325, complete genome.\|SAOUHSC_00004 | 0.889871 | 0.00025 | 0.02705 |
| 1. CDS\|GI:88196463;GeneID:3921268\|GenBank\|0904\|NC_007795\|2607117-2607788\|NAD(P)H-flavin oxidoreductase, putative\|\|Staphylococcus aureus subsp. aureus NCTC 8325, complete genome.\|SAOUHSC_02829 | 0.838626 | 0.000268 | 0.02806 |
| 1. CDS\|GI:88195057;GeneID:3920192\|GenBank\|0904\|NC_007795\|1270122-1271543\|catalase\|\|Staphylococcus aureus subsp. aureus NCTC 8325, complete genome.\|SAOUHSC_01327 | 0.755152 | 0.000286 | 0.029301 |
| 1. CDS\|GI:88195501;GeneID:3920442\|GenBank\|0904\|NC_007795\|1701494-1702762\|isocitrate dehydrogenase\|Converts isocitrate to alpha ketoglutarate\|Staphylococcus aureus subsp. aureus NCTC 8325, complete genome.\|SAOUHSC_01801 | 1.173667 | 0.000294 | 0.029622 |
| 1. CDS\|GI:88193828;GeneID:3919178\|GenBank\|0904\|NC_007795\|5034-6968\|DNA gyrase, B subunit\|\|Staphylococcus aureus subsp. aureus NCTC 8325, complete genome.\|SAOUHSC_00005 | 0.785884 | 0.000389 | 0.031095 |
| 1. CDS\|GI:88195066;GeneID:3920201\|GenBank\|0904\|NC_007795\|1276664-1278541\|transketolase\|\|Staphylococcus aureus subsp. aureus NCTC 8325, complete genome.\|SAOUHSC_01337 | 0.798083 | 0.000458 | 0.03561 |
| 1. CDS\|GI:88194750;GeneID:3920394\|GenBank\|0904\|NC_007795\|964093-967863\|bifunctional autolysin precursor, putative\|\|Staphylococcus aureus subsp. aureus NCTC 8325, complete genome.\|SAOUHSC_00994 | 0.978469 | 0.000519 | 0.038811 |
| 1. CDS\|GI:88194757;GeneID:3920401\|GenBank\|0904\|NC_007795\|973478-975466\|quinol oxidase, subunit I\|\|Staphylococcus aureus subsp. aureus NCTC 8325, complete genome.\|SAOUHSC_01001 | 0.798041 | 0.00065 | 0.038969 |
| 1. CDS\|GI:88195217;GeneID:3919044\|GenBank\|0904\|NC_007795\|1453688-1455148\|elastin binding protein\|\|Staphylococcus aureus subsp. aureus NCTC 8325, complete genome.\|SAOUHSC_01501 | 0.690287 | 0.000569 | 0.039072 |
| 1. CDS\|GI:88195390;GeneID:3921796\|GenBank\|0904\|NC_007795\|1594514-1595140\|co-chaperone GrpE\|\|Staphylococcus aureus subsp. aureus NCTC 8325, complete genome.\|SAOUHSC_01684 | 1.656685 | 0.000668 | 0.041318 |
| 1. CDS\|GI:88195075;GeneID:3920053\|GenBank\|0904\|NC_007795\|1287161-1289866\|aconitate hydratase\|Catalyzes the conversion of citrate to isocitrate\|Staphylococcus aureus subsp. aureus NCTC 8325, complete genome.\|SAOUHSC_01347 | 0.611416 | 0.000681 | 0.043356 |
| 1. CDS\|GI:88195010;GeneID:3919930\|GenBank\|0904\|NC_007795\|1234935-1236608\|aerobic glycerol-3-phosphate dehydrogenase\|conserved hypothetical protein\|Staphylococcus aureus subsp. aureus NCTC 8325, complete genome.\|SAOUHSC_01278 | 0.609469 | 0.001037 | 0.04643 |
| 1. CDS\|GI:88195324;GeneID:3920029\|GenBank\|0904\|NC_007795\|1536907-1537899\|2-oxoisovalerate dehydrogenase, E1 component, alpha subunit, putative\|\|Staphylococcus aureus subsp. aureus NCTC 8325, complete genome.\|SAOUHSC_01613 | 0.60273 | 0.000876 | 0.046735 |
| 1. CDS\|GI:88195323;GeneID:3920028\|GenBank\|0904\|NC_007795\|1535924-1536907\|2-oxoisovalerate dehydrogenase, E1 component, beta subunit, putative\|\|Staphylococcus aureus subsp. aureus NCTC 8325, complete genome.\|SAOUHSC_01612 | 0.706462 | 0.001277 | 0.047433 |
| 1. CDS\|GI:88195132;GeneID:3920696\|GenBank\|0904\|NC_007795\|1346759-1347028\|acylphosphatase\|\|Staphylococcus aureus subsp. aureus NCTC 8325, complete genome.\|SAOUHSC_01406 | 0.831033 | 0.000902 | 0.049099 |
| 1. CDS\|GI:88194558;GeneID:3919361\|GenBank\|0904\|NC_007795\|780410-781927\|phosphoglyceromutase\|catalyzes the interconversion of 2-phosphoglycerate and 3-phosphoglycerate\|Staphylococcus aureus subsp. aureus NCTC 8325, complete genome.\|SAOUHSC_00798 | 0.583166 | 0.001129 | 0.052073 |
| 1. CDS\|GI:88194555;GeneID:3919358\|GenBank\|0904\|NC_007795\|777185-778195\|glyceraldehyde-3-phosphate dehydrogenase, type I\|\|Staphylococcus aureus subsp. aureus NCTC 8325, complete genome.\|SAOUHSC_00795 | 0.727307 | 0.001089 | 0.052946 |
| 1. CDS\|GI:88195116;GeneID:3920680\|GenBank\|0904\|NC_007795\|1331363-1332346\|phosphate ABC transporter, periplasmic phosphate-binding protein, putative\|\|Staphylococcus aureus subsp. aureus NCTC 8325, complete genome.\|SAOUHSC_01389 | 1.161089 | 0.001216 | 0.053339 |
| 1. CDS\|GI:88194286;GeneID:3920417\|GenBank\|0904\|NC_007795\|505226-507682\|endopeptidase, putative\|\|Staphylococcus aureus subsp. aureus NCTC 8325, complete genome.\|SAOUHSC_00505 | 0.903263 | 0.001218 | 0.05487 |
| 1. CDS\|GI:88194761;GeneID:3920405\|GenBank\|0904\|NC_007795\|978261-979121\|FolD bifunctional protein, putative\|\|Staphylococcus aureus subsp. aureus NCTC 8325, complete genome.\|SAOUHSC_01007 | 0.608987 | 0.001894 | 0.062142 |
| 1. CDS\|GI:88194005;GeneID:3920351\|GenBank\|0904\|NC_007795\|214246-215430\|acetyl-CoA acetyltransferase, putative\|\|Staphylococcus aureus subsp. aureus NCTC 8325, complete genome.\|SAOUHSC_00195 | 0.490782 | 0.002137 | 0.062226 |
| 1. CDS\|GI:88194756;GeneID:3920400\|GenBank\|0904\|NC_007795\|972883-973488\|cytochrome c oxidase subunit III, putative\|\|Staphylococcus aureus subsp. aureus NCTC 8325, complete genome.\|SAOUHSC_01000 | 0.595391 | 0.002464 | 0.062628 |
| 1. CDS\|GI:88194758;GeneID:3920402\|GenBank\|0904\|NC_007795\|975466-976566\|quinol oxidase AA3, subunit II, putative\|\|Staphylococcus aureus subsp. aureus NCTC 8325, complete genome.\|SAOUHSC_01002 | 1.001607 | 0.001906 | 0.063384 |
| 1. CDS\|GI:88195499;GeneID:3920440\|GenBank\|0904\|NC_007795\|1698739-1700400\|histidine kinase\|Histidine kinase-, DNA gyrase B-, and HSP90-like ATPase domain protein\|Staphylococcus aureus subsp. aureus NCTC 8325, complete genome.\|SAOUHSC_01799 | 0.591337 | 0.00163 | 0.063574 |
| 1. CDS\|GI:88195391;GeneID:3921797\|GenBank\|0904\|NC_007795\|1595172-1596149\|heat-inducible transcription repressor HrcA\|\|Staphylococcus aureus subsp. aureus NCTC 8325, complete genome.\|SAOUHSC_01685 | 1.503368 | 0.001834 | 0.0657 |
| 1. CDS\|GI:88195479;GeneID:3919696\|GenBank\|0904\|NC_007795\|1677874-1679136\|ATP-dependent protease ATP-binding subunit ClpX\|binds and unfolds substrates as part of the ClpXP protease\|Staphylococcus aureus subsp. aureus NCTC 8325, complete genome.\|SAOUHSC_01778 | 0.69154 | 0.002279 | 0.065841 |
| 1. CDS\|GI:88194162;GeneID:3919783\|GenBank\|0904\|NC_007795\|369729-371252\|alkyl hydroperoxide reductase subunit F\|alkyl hydroperoxide reductase, subunit F, putative\|Staphylococcus aureus subsp. aureus NCTC 8325, complete genome.\|SAOUHSC_00364 | 0.53856 | 0.002449 | 0.066896 |
| 1. CDS\|GI:88196625;GeneID:3921488\|GenBank\|0904\|NC_007795\|2779036-2781078\|lipase\|\|Staphylococcus aureus subsp. aureus NCTC 8325, complete genome.\|SAOUHSC_03006 | 0.530124 | 0.002161 | 0.067016 |
| 1. CDS\|GI:88195154;GeneID:3920211\|GenBank\|0904\|NC_007795\|1367604-1368104\|phosphotransferase system enzyme IIA, putative\|\|Staphylococcus aureus subsp. aureus NCTC 8325, complete genome.\|SAOUHSC_01430 | 0.700731 | 0.002282 | 0.071053 |
| 1. CDS\|GI:88195298;GeneID:3920003\|GenBank\|0904\|NC_007795\|1514324-1515061\|ribosomal large subunit pseudouridine synthase B\|ribosomal large subunit pseudouridine synthase B, putative\|Staphylococcus aureus subsp. aureus NCTC 8325, complete genome.\|SAOUHSC_01587 | 0.512677 | 0.002884 | 0.071802 |
| 1. CDS\|GI:88194936;GeneID:3919330\|GenBank\|0904\|NC_007795\|1149649-1150575\|malonyl CoA-acyl carrier protein transacylase\|\|Staphylococcus aureus subsp. aureus NCTC 8325, complete genome.\|SAOUHSC_01198 | 0.61821 | 0.002919 | 0.073713 |
| 1. CDS\|GI:88194891;GeneID:3920709\|GenBank\|0904\|NC_007795\|1100157-1101569\|cell division protein, putative\|\|Staphylococcus aureus subsp. aureus NCTC 8325, complete genome.\|SAOUHSC_01149 | 0.768014 | 0.003115 | 0.074053 |
| 1. CDS\|GI:88194285;GeneID:3920416\|GenBank\|0904\|NC_007795\|504205-505212\|ATP:guanido phosphotransferase\|ATP:guanido phosphotransferase, C-terminal catalytic domain protein\|Staphylococcus aureus subsp. aureus NCTC 8325, complete genome.\|SAOUHSC_00504 | 0.744012 | 0.003062 | 0.074117 |
| 1. CDS\|GI:88195377;GeneID:3920082\|GenBank\|0904\|NC_007795\|1582690-1583094\|cytidine deaminase\|\|Staphylococcus aureus subsp. aureus NCTC 8325, complete genome.\|SAOUHSC_01670 | 0.47266 | 0.003736 | 0.074376 |
| 1. CDS\|GI:88194852;GeneID:3920748\|GenBank\|0904\|NC_007795\|1068153-1068953\|glutamate racemase\|converts L-glutamate to D-glutamate, a component of peptidoglycan\|Staphylococcus aureus subsp. aureus NCTC 8325, complete genome.\|SAOUHSC_01106 | 0.461484 | 0.002749 | 0.074935 |
| 1. CDS\|GI:88195325;GeneID:3920030\|GenBank\|0904\|NC_007795\|1537915-1539336\|dihydrolipoamide dehydrogenase\|\|Staphylococcus aureus subsp. aureus NCTC 8325, complete genome.\|SAOUHSC_01614 | 0.605102 | 0.002873 | 0.076137 |
| 1. CDS\|GI:88194163;GeneID:3919784\|GenBank\|0904\|NC_007795\|371268-371837\|alkyl hydroperoxide reductase subunit C\|alkyl hydroperoxide reductase\|Staphylococcus aureus subsp. aureus NCTC 8325, complete genome.\|SAOUHSC_00365 | 0.932129 | 0.002892 | 0.076626 |
| 1. CDS\|GI:88195378;GeneID:3920083\|GenBank\|0904\|NC_007795\|1583105-1583449\|diacylglycerol kinase, putative\|\|Staphylococcus aureus subsp. aureus NCTC 8325, complete genome.\|SAOUHSC_01671 | 0.523509 | 0.003306 | 0.077454 |
| 1. CDS\|GI:88195935;GeneID:3919140\|GenBank\|0904\|NC_007795\|2096006-2096632\|accessory gene regulator protein A\|\|Staphylococcus aureus subsp. aureus NCTC 8325, complete genome.\|SAOUHSC_02265 | 0.48879 | 0.003496 | 0.077461 |
| 1. CDS\|GI:88194781;GeneID:3920288\|GenBank\|0904\|NC_007795\|998122-998388\|phosphocarrier protein HPr\|phosphocarrier protein hpr, putative\|Staphylococcus aureus subsp. aureus NCTC 8325, complete genome.\|SAOUHSC_01028 | 0.731024 | 0.003276 | 0.081001 |
| 1. CDS\|GI:88195497;GeneID:3920438\|GenBank\|0904\|NC_007795\|1693829-1696459\|DNA polymerase I\|\|Staphylococcus aureus subsp. aureus NCTC 8325, complete genome.\|SAOUHSC_01797 | 0.683587 | 0.005614 | 0.082119 |
| 1. CDS\|GI:88195326;GeneID:3920031\|GenBank\|0904\|NC_007795\|1539487-1541166\|DNA repair protein RecN\|\|Staphylococcus aureus subsp. aureus NCTC 8325, complete genome.\|SAOUHSC_01615 | 0.696687 | 0.004571 | 0.084198 |
| 1. CDS\|GI:88194961;GeneID:3920253\|GenBank\|0904\|NC_007795\|1177666-1178211\|ATP-dependent protease peptidase subunit\|heat shock protein involved in degradation of misfolded proteins\|Staphylococcus aureus subsp. aureus NCTC 8325, complete genome.\|SAOUHSC_01225 | 0.51023 | 0.005822 | 0.08437 |
| 1. CDS\|GI:88195370;GeneID:3920075\|GenBank\|0904\|NC_007795\|1575828-1577627\|DNA primase\|DNA primase, putative\|Staphylococcus aureus subsp. aureus NCTC 8325, complete genome.\|SAOUHSC_01663 | 0.908928 | 0.004269 | 0.086023 |
| 1. CDS\|GI:88196015;GeneID:3919395\|GenBank\|0904\|NC_007795\|2174506-2175633\|UDP-GlcNAc 2-epimerase\|UDP-N-acetylglucosamine 2-epimerase\|Staphylococcus aureus subsp. aureus NCTC 8325, complete genome.\|SAOUHSC_02352 | 0.631203 | 0.005739 | 0.087021 |
| 1. CDS\|GI:88196016;GeneID:3919396\|GenBank\|0904\|NC_007795\|2175657-2176286\|uracil phosphoribosyltransferase\|\|Staphylococcus aureus subsp. aureus NCTC 8325, complete genome.\|SAOUHSC_02353 | 0.652216 | 0.006801 | 0.087716 |
| 1. CDS\|GI:88195967;GeneID:3919172\|GenBank\|0904\|NC_007795\|2133600-2134388\|sigmaB regulation protein RsbU, putative\|\|Staphylococcus aureus subsp. aureus NCTC 8325, complete genome.\|SAOUHSC_02301 | 0.667581 | 0.004648 | 0.088762 |
| 1. CDS\|GI:88195186;GeneID:3919911\|GenBank\|0904\|NC_007795\|1424647-1425306\|endonuclease III, putative\|\|Staphylococcus aureus subsp. aureus NCTC 8325, complete genome.\|SAOUHSC_01469 | 0.550133 | 0.006671 | 0.08976 |
| 1. CDS\|GI:88195322;GeneID:3920027\|GenBank\|0904\|NC_007795\|1534637-1535911\|2-oxoisovalerate dehydrogenase, E2 component, dihydrolipoamide acetyltransferase, putative\|\|Staphylococcus aureus subsp. aureus NCTC 8325, complete genome.\|SAOUHSC_01611 | 0.805491 | 0.005686 | 0.090557 |
| 1. CDS\|GI:88194960;GeneID:3920252\|GenBank\|0904\|NC_007795\|1176773-1177669\|site-specific recombinase, putative\|\|Staphylococcus aureus subsp. aureus NCTC 8325, complete genome.\|SAOUHSC_01224 | 0.66207 | 0.00515 | 0.09107 |
| 1. CDS\|GI:88195112;GeneID:3920794\|GenBank\|0904\|NC_007795\|1328429-1329280\|phosphate ABC transporter ATP-binding protein, putative\|\|Staphylococcus aureus subsp. aureus NCTC 8325, complete genome.\|SAOUHSC_01385 | 0.614949 | 0.006969 | 0.093908 |
| 1. CDS\|GI:88195113;GeneID:3920677\|GenBank\|0904\|NC_007795\|1329327-1330244\|phosphate ABC transporter, permease protein, putative\|\|Staphylococcus aureus subsp. aureus NCTC 8325, complete genome.\|SAOUHSC_01386 | 0.605278 | 0.007637 | 0.097158 |
| 1. CDS\|GI:88195968;GeneID:3919173\|GenBank\|0904\|NC_007795\|2134363-2134653\|sigmaB regulation protein RsbU, putative\|\|Staphylococcus aureus subsp. aureus NCTC 8325, complete genome.\|SAOUHSC_02302 | 0.901276 | 0.007303 | 0.097327 |
| 1. CDS\|GI:88195354;GeneID:3920098\|GenBank\|0904\|NC_007795\|1561291-1562277\|glucocaase\|glucokinase, putative\|Staphylococcus aureus subsp. aureus NCTC 8325, complete genome.\|SAOUHSC_01646 | 0.47617 | 0.00788 | 0.098329 |
| 1. CDS\|GI:88196225;GeneID:3921578\|GenBank\|0904\|NC_007795\|2371792-2374746\|formate dehydrogenase, alpha subunit, putative\|\|Staphylococcus aureus subsp. aureus NCTC 8325, complete genome.\|SAOUHSC_02582 | 0.649743 | 0.009411 | 0.09888 |
| 1. CDS\|GI:88196183;GeneID:3921126\|GenBank\|0904\|NC_007795\|2336046-2337068\|molybdenum cofactor biosynthesis protein A\|molybdopterin cofactor biosynthesis protein A, putative\|Staphylococcus aureus subsp. aureus NCTC 8325, complete genome.\|SAOUHSC_02536 | 0.470548 | 0.007836 | 0.099023 |
| 1. CDS\|GI:88195511;GeneID:3919281\|GenBank\|0904\|NC_007795\|1714657-1717854\|DNA polymerase III alpha subunit superfamily protein\|DNA polymerase III, alpha subunit superfamily\|Staphylococcus aureus subsp. aureus NCTC 8325, complete genome.\|SAOUHSC_01811 | 0.483159 | 0.00864 | 0.099681 |
| 1. CDS\|GI:88196669;GeneID:3921318\|GenBank\|0904\|NC_007795\|2821010-2821147\|50S ribosomal protein L34\|in Escherichia coli transcription of this gene is enhanced by polyamines\|Staphylococcus aureus subsp. aureus NCTC 8325, complete genome.\|SAOUHSC_03055 | -0.38392 | 0.00802 | 0.088177 |
| 1. CDS\|GI:88196320;GeneID:3921242\|GenBank\|0904\|NC_007795\|2462342-2463787\|nitrate reductase, beta subunit\|\|Staphylococcus aureus subsp. aureus NCTC 8325, complete genome.\|SAOUHSC_02680 | -0.42868 | 0.007863 | 0.095519 |
| 1. CDS\|GI:88195922;GeneID:3919671\|GenBank\|0904\|NC_007795\|2085259-2085849\|phage terminase, small subunit, putative\|\|Staphylococcus aureus subsp. aureus NCTC 8325, complete genome.\|SAOUHSC_02250 | -0.38687 | 0.00684 | 0.096262 |
| 1. CDS\|GI:88194158;GeneID:3919779\|GenBank\|0904\|NC_007795\|367373-367954\|phosphoglycerate mutase family protein\|\|Staphylococcus aureus subsp. aureus NCTC 8325, complete genome.\|SAOUHSC_00359 | -0.32644 | 0.008244 | 0.098865 |
| 1. CDS\|GI:88196504;GeneID:3921545\|GenBank\|0904\|NC_007795\|2647869-2648075\|cation transporter E1-E2 family ATPase\|cation-transporting ATPase, E1-E2 family, putative\|Staphylococcus aureus subsp. aureus NCTC 8325, complete genome.\|SAOUHSC_02874 | -0.40616 | 0.010771 | 0.098961 |

| **Table S2: DEGs between 144 hr biofilm growth vs. planktonic** | | | |
| --- | --- | --- | --- |
| GENE_INFO | logFC | P.Value | adj.P.Val |
| 1. CDS\|GI:88195391;GeneID:3921797\|GenBank\|0904\|NC_007795\|1595172-1596149\|heat-inducible transcription repressor HrcA\|\|Staphylococcus aureus subsp. aureus NCTC 8325, complete genome.\|SAOUHSC_01685 | 1.280553 | 1.22E-05 | 0.006306 |
| 1. CDS\|GI:88195390;GeneID:3921796\|GenBank\|0904\|NC_007795\|1594514-1595140\|co-chaperone GrpE\|\|Staphylococcus aureus subsp. aureus NCTC 8325, complete genome.\|SAOUHSC_01684 | 1.302759 | 7.78E-05 | 0.019986 |
| 1. CDS\|GI:88194286;GeneID:3920417\|GenBank\|0904\|NC_007795\|505226-507682\|endopeptidase, putative\|\|Staphylococcus aureus subsp. aureus NCTC 8325, complete genome.\|SAOUHSC_00505 | 0.501858 | 9.98E-05 | 0.020805 |
| 1. CDS\|GI:88194953;GeneID:3919482\|GenBank\|0904\|NC_007795\|1166886-1168052\|succinyl-CoA synthetase subunit beta\|catalyzes the interconversion of succinyl-CoA and succinate\|Staphylococcus aureus subsp. aureus NCTC 8325, complete genome.\|SAOUHSC_01216 | 0.422031 | 0.000138 | 0.031781 |
| 1. CDS\|GI:88194652;GeneID:3921741\|GenBank\|0904\|NC_007795\|859017-860261\|glutamate dehydrogenase, NAD-specific, putative\|\|Staphylococcus aureus subsp. aureus NCTC 8325, complete genome.\|SAOUHSC_00895 | 0.525218 | 0.000173 | 0.033546 |
| 1. CDS\|GI:88194651;GeneID:3921740\|GenBank\|0904\|NC_007795\|857718-858908\|ornithine--oxo-acid transaminase\|ornithine aminotransferase, putative\|Staphylococcus aureus subsp. aureus NCTC 8325, complete genome.\|SAOUHSC_00894 | 0.569417 | 0.000683 | 0.068405 |
| 1. CDS\|GI:88195931;GeneID:3919680\|GenBank\|0904\|NC_007795\|2093504-2093638\|delta-hemolysin precursor\|\|Staphylococcus aureus subsp. aureus NCTC 8325, complete genome.\|SAOUHSC_02260 | -1.82973 | 4.69E-07 | 0.001287de |
| 1. CDS\|GI:88196493;GeneID:3921534\|GenBank\|0904\|NC_007795\|2633834-2635939\|ATP-dependent Clp protease, ATP-binding subunit ClpC, putative\|\|Staphylococcus aureus subsp. aureus NCTC 8325, complete genome.\|SAOUHSC_02862 | -0.67926 | 1.02E-06 | 0.001469 |
| 1. CDS\|GI:88194572;GeneID:3919375\|GenBank\|0904\|NC_007795\|792986-795769\|clumping factor\|\|Staphylococcus aureus subsp. aureus NCTC 8325, complete genome.\|SAOUHSC_00812 | -0.78833 | 7.38E-06 | 0.004114 |
| 1. CDS\|GI:88196118;GeneID:3919029\|GenBank\|0904\|NC_007795\|2289534-2289959\|truncated MHC class II analog protein\|\|Staphylococcus aureus subsp. aureus NCTC 8325, complete genome.\|SAOUHSC_02466 | -1.22934 | 1.35E-05 | 0.005573 |

| **Table S3:DEGs between 144 hr biofilm growth vs. 48 hr biofilm growth** | | | |
| --- | --- | --- | --- |
| GENE_INFO | logFC | P.Value | adj.P.Val |
| 1. CDS\|GI:88195931;GeneID:3919680\|GenBank\|0904\|NC_007795\|2093504-2093638\|delta-hemolysin precursor\|\|Staphylococcus aureus subsp. aureus NCTC 8325, complete genome.\|SAOUHSC_02260 | -1.9269 | 1.85E-06 | 0.006731 |
| 1. CDS\|GI:88195932;GeneID:3919137\|GenBank\|0904\|NC_007795\|2093874-2094443\|accessory gene regulator protein B\|\|Staphylococcus aureus subsp. aureus NCTC 8325, complete genome.\|SAOUHSC_02261 | -1.44715 | 4.03E-05 | 0.04889 |
| 1. CDS\|GI:88195934;GeneID:3919139\|GenBank\|0904\|NC_007795\|2094653-2095897\|accessory gene regulator protein C\|\|Staphylococcus aureus subsp. aureus NCTC 8325, complete genome.\|SAOUHSC_02264 | -1.40917 | 7.87E-05 | 0.060421 |
| 1. CDS\|GI:88195361;GeneID:3920105\|GenBank\|0904\|NC_007795\|1567139-1567738\|superoxide dismutase, Mn, putative\|\|Staphylococcus aureus subsp. aureus NCTC 8325, complete genome.\|SAOUHSC_01653 | -1.16044 | 0.000153 | 0.069928 |
| 1. CDS\|GI:88196118;GeneID:3919029\|GenBank\|0904\|NC_007795\|2289534-2289959\|truncated MHC class II analog protein\|\|Staphylococcus aureus subsp. aureus NCTC 8325, complete genome.\|SAOUHSC_02466 | -1.03296 | 0.000132 | 0.071871 |
